# Supplementary material for: CK2 Activity Mediates the Aggressive Molecular Signature of Glioblastoma Multiforme by Inducing Nerve/Glial Antigen (NG)2 Expression
Source: Cancers (Basel). 2021 Apr 2;13(7):1678. doi: 10.3390/cancers13071678 (PMC8037969; doi:10.3390/cancers13071678)
Supplement: Supplementary file 1 [file cancers-13-01678-s001.pdf]

Supplementary Materials: CK2 Activity Mediates the Aggressive Molecular Signature of Glioblastoma Multiforme by Inducing Nerve/Glial Antigen (NG)2 Expression

Beate M. Schmitt, Anne S. Boewe, Claudia Götz, Stephan E. Philipp, Steffi Urbschat, Joachim Oertel, Michael D. Menger, Matthias W. Laschke and Emmanuel Ampofo

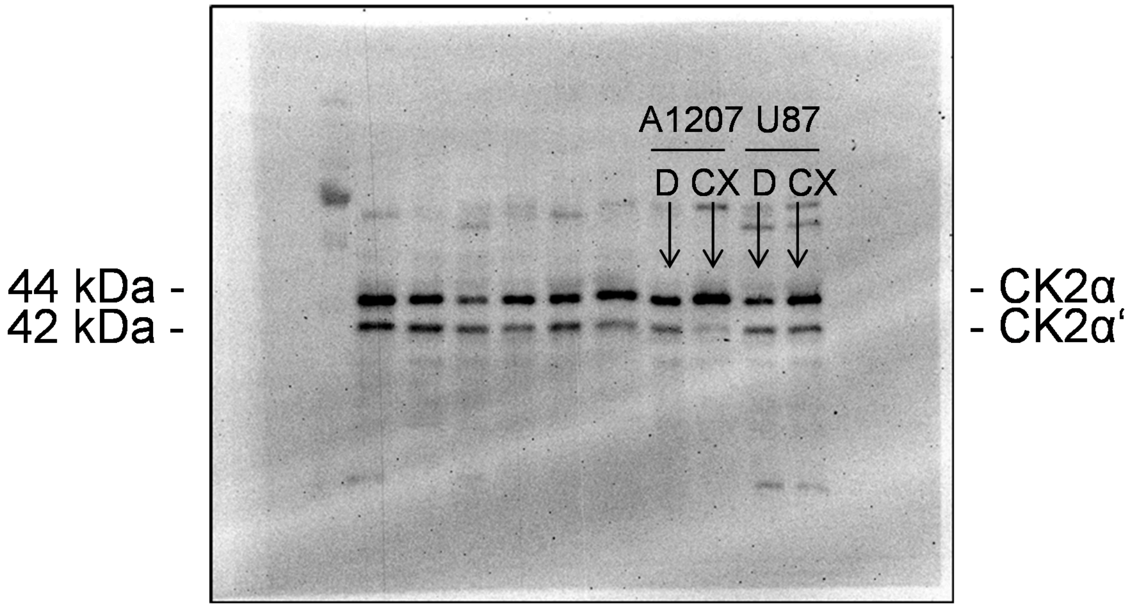

Figure S1. CK2 inhibition reduces NG2 expression in human GBM cell lines. A1207 and U87 cells were treated with vehicle (DMSO) or CX-4945 (10 μM) for 72 h. The cells were lysed and the expression of CK2α and CK2α′ was analyzed by western blot.

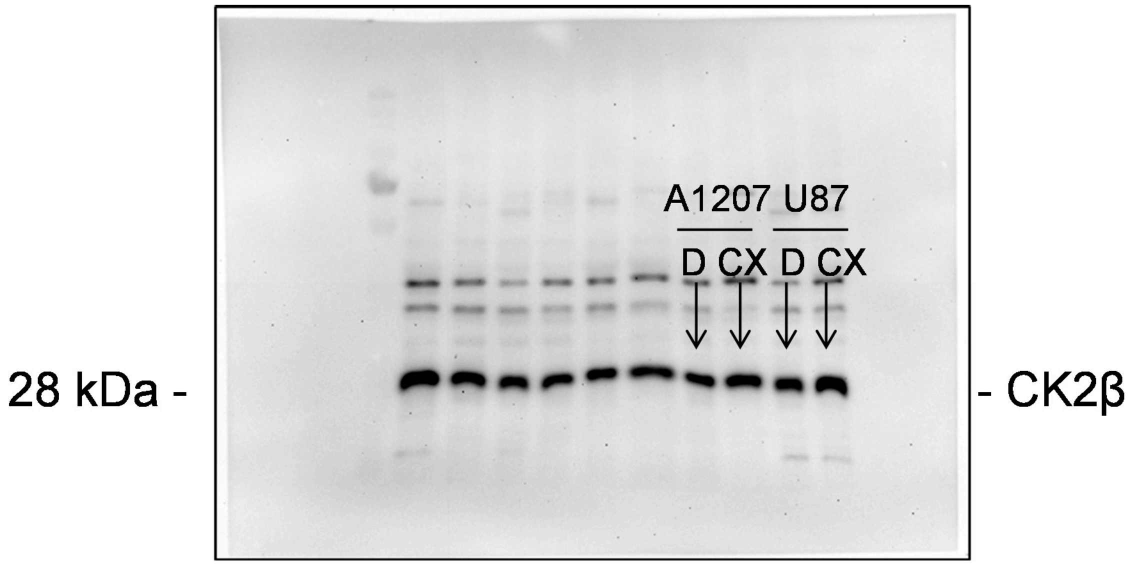

Figure S2. CK2 inhibition reduces NG2 expression in human GBM cell lines. A1207 and U87 cells were treated with vehicle (DMSO) or CX-4945 (10 μM) for 72 h. The cells were lysed and the expression of CK2β was analyzed by western blot.

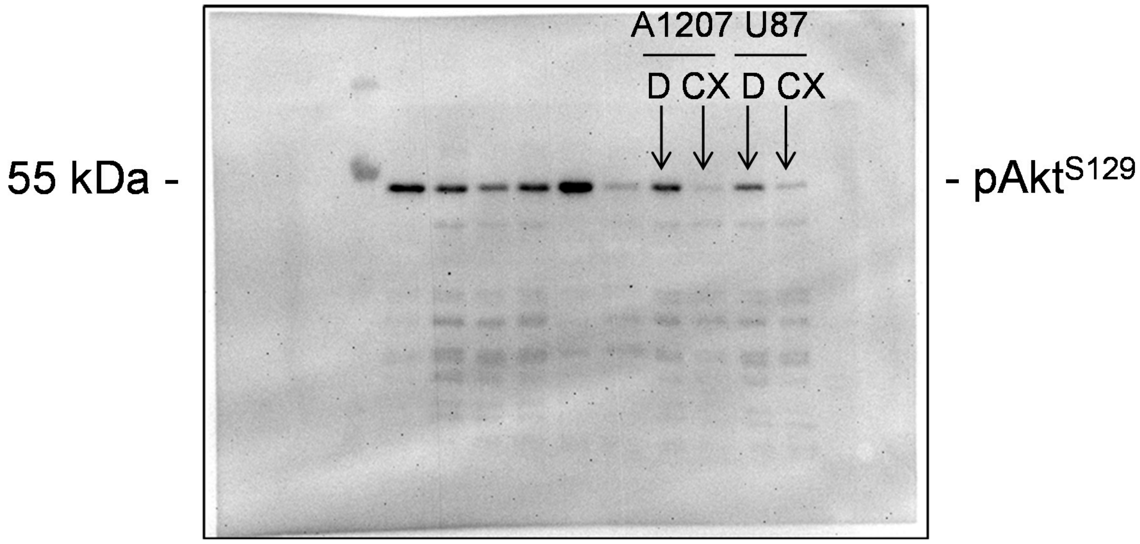

Figure S3. CK2 inhibition reduces NG2 expression in human GBM cell lines. A1207 and U87 cells were treated with vehicle (DMSO) or CX-4945 (10 μM) for 72 h. The cells were lysed and the expression of pAkt^{S129} was analyzed by western blot.

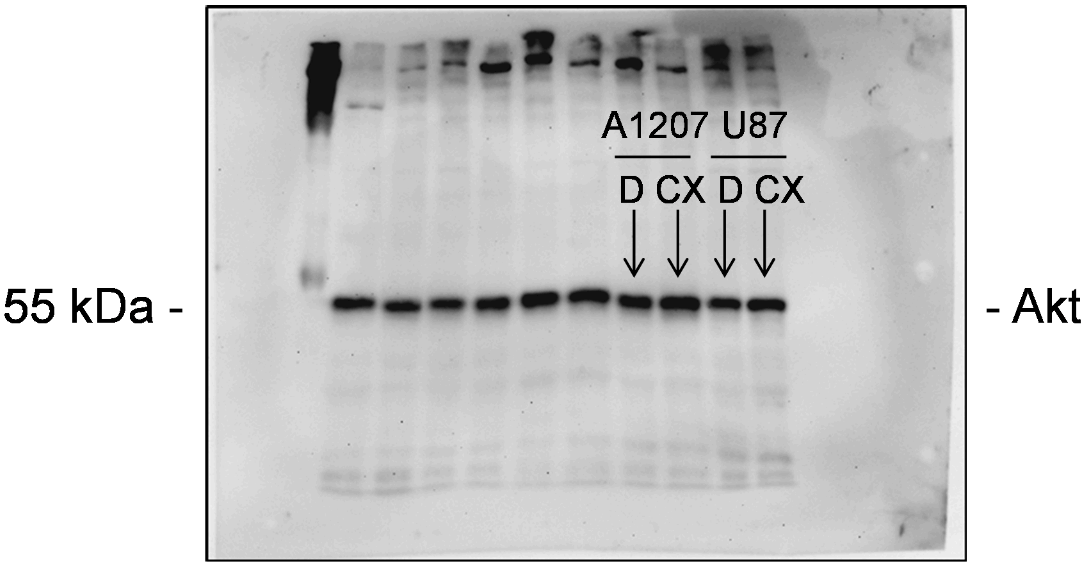

Figure S4. CK2 inhibition reduces NG2 expression in human GBM cell lines. A1207 and U87 cells were treated with vehicle (DMSO) or CX-4945 (10 μM) for 72 h. The cells were lysed and the expression of Akt was analyzed by western blot.

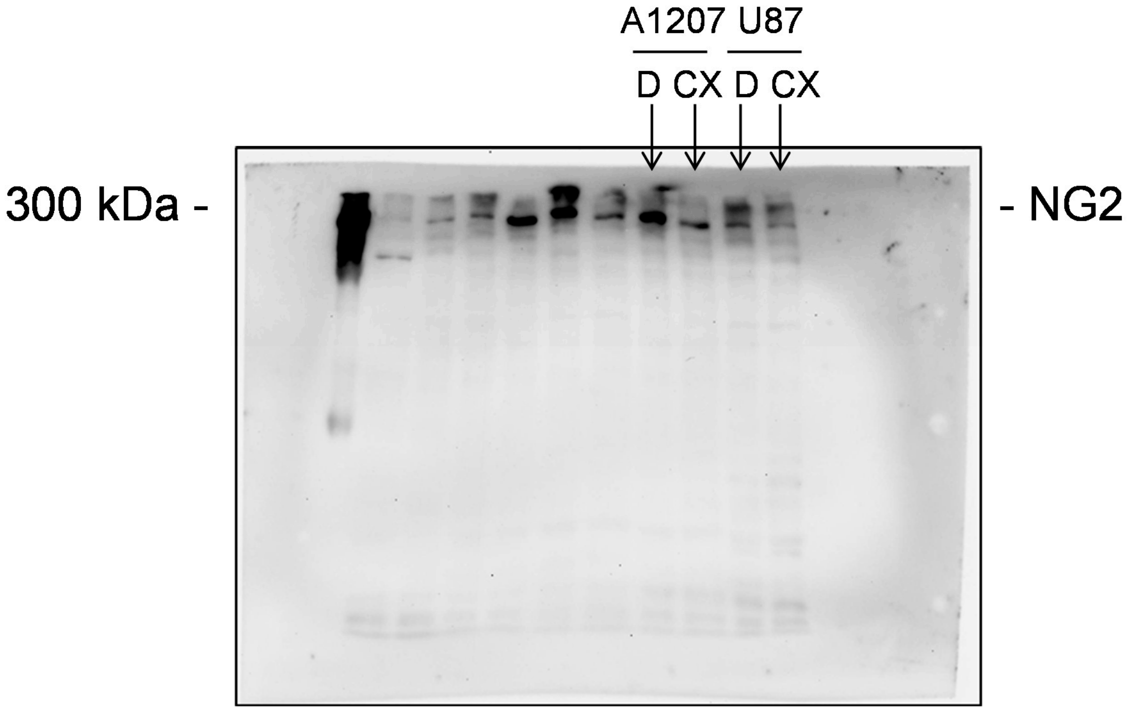

Figure S5. CK2 inhibition reduces NG2 expression in human GBM cell lines. A1207 and U87 cells were treated with vehicle (DMSO) or CX-4945 (10 μM) for 72 h. The cells were lysed and the expression of NG2 was analyzed by western blot.

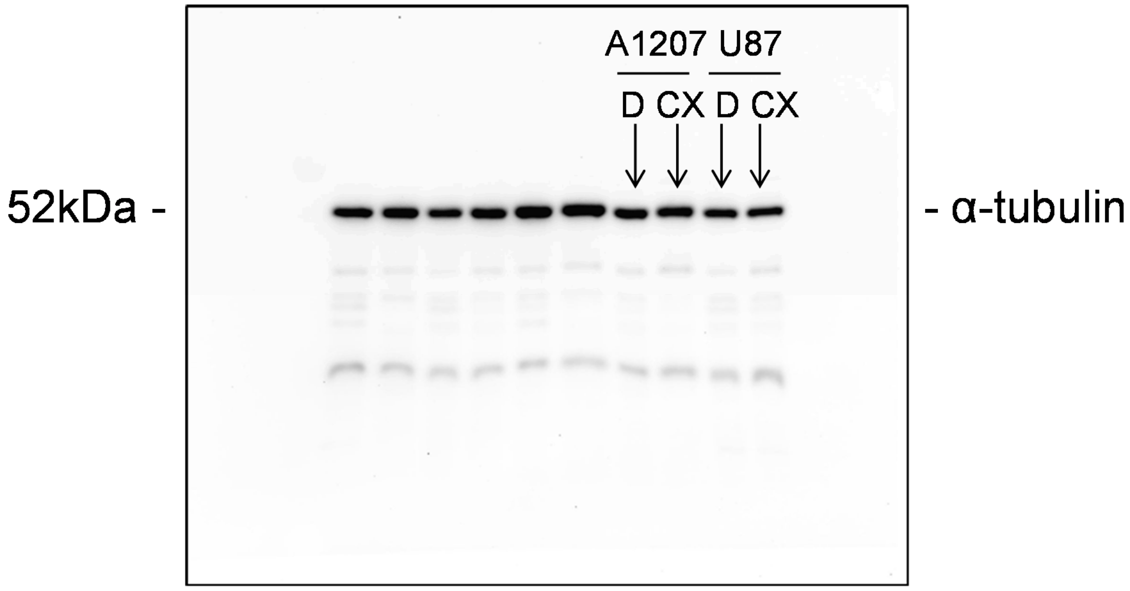

Figure S6. CK2 inhibition reduces NG2 expression in human GBM cell lines. A1207 and U87 cells were treated with vehicle (DMSO) or CX-4945 (10 μM) for 72 h. The cells were lysed and the expression of α-tubulin (as loading control) was analyzed by western blot.

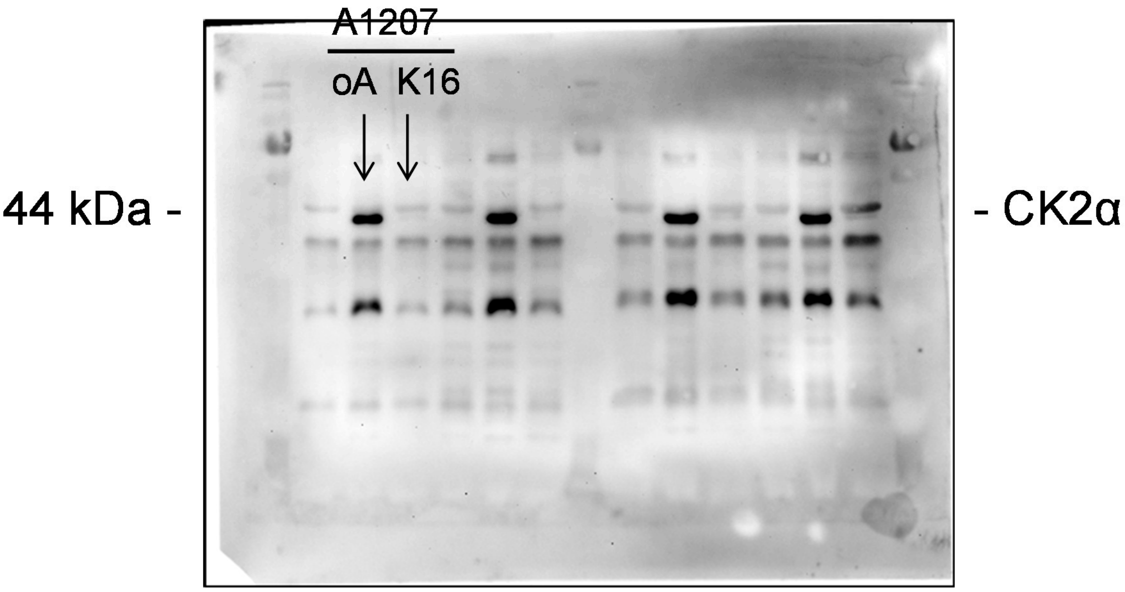

Figure S7. CK2 inhibition reduces NG2 expression in human GBM cell lines. A1207 wild type and CK2α KO cells were lysed and the expression of CK2α was analyzed by western blot.

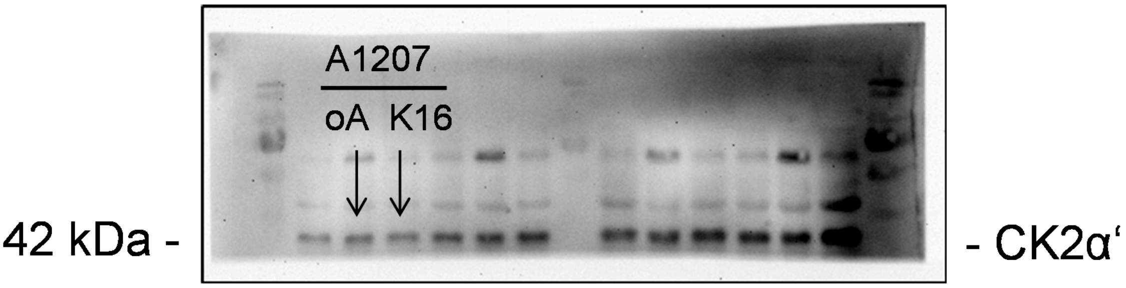

Figure S8. CK2 inhibition reduces NG2 expression in human GBM cell lines. A1207 wild type and CK2α KO cells were lysed and the expression of CK2α' was analyzed by western blot.

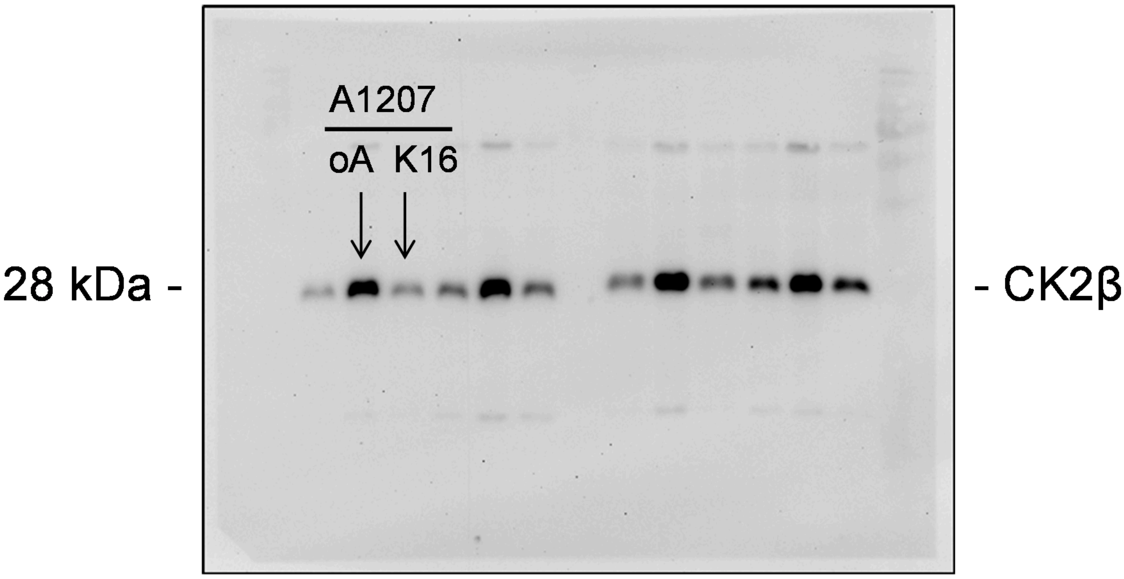

Figure S9. CK2 inhibition reduces NG2 expression in human GBM cell lines. A1207 wild type and CK2α KO cells were lysed and the expression of CK2β was analyzed by western blot.

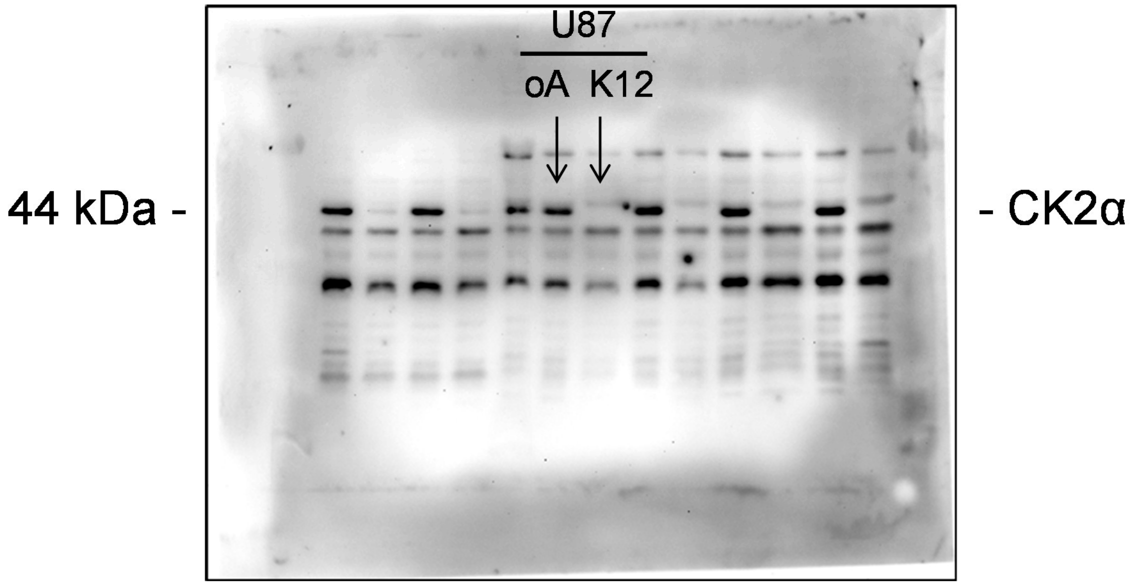

Figure S10. CK2 inhibition reduces NG2 expression in human GBM cell lines. U87 wild type and CK2*α* KO cells were lysed and the expression of CK2*α* was analyzed by western blot.

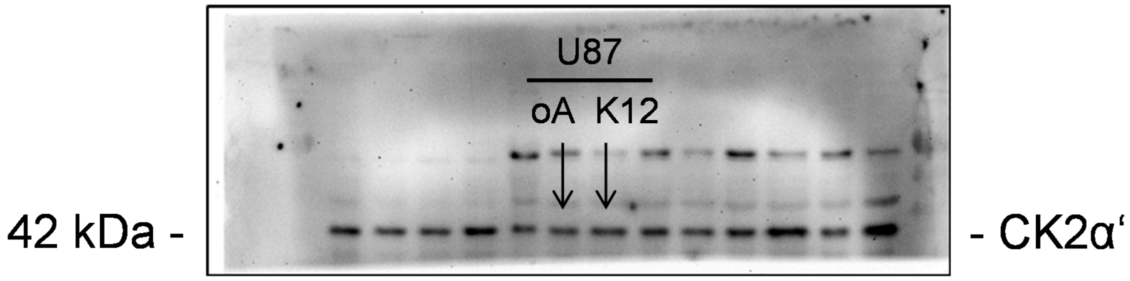

Figure S11. CK2 inhibition reduces NG2 expression in human GBM cell lines. U87 wild type and CK2*α* KO cells were lysed and the expression of CK2*α′* was analyzed by western blot.

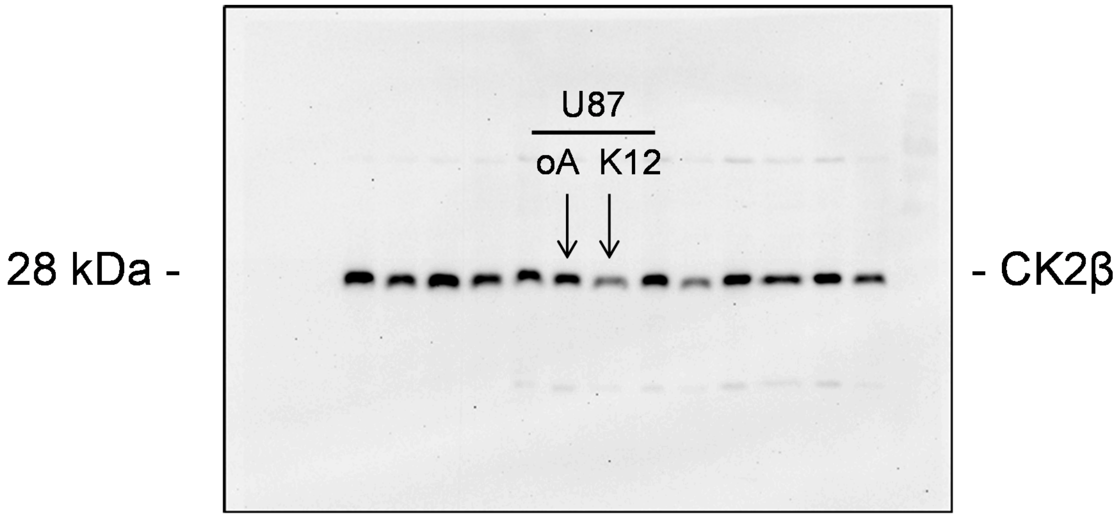

Figure S12. CK2 inhibition reduces NG2 expression in human GBM cell lines. U87 wild type and CK2*α* KO cells were lysed and the expression of CK2β was analyzed by western blot.

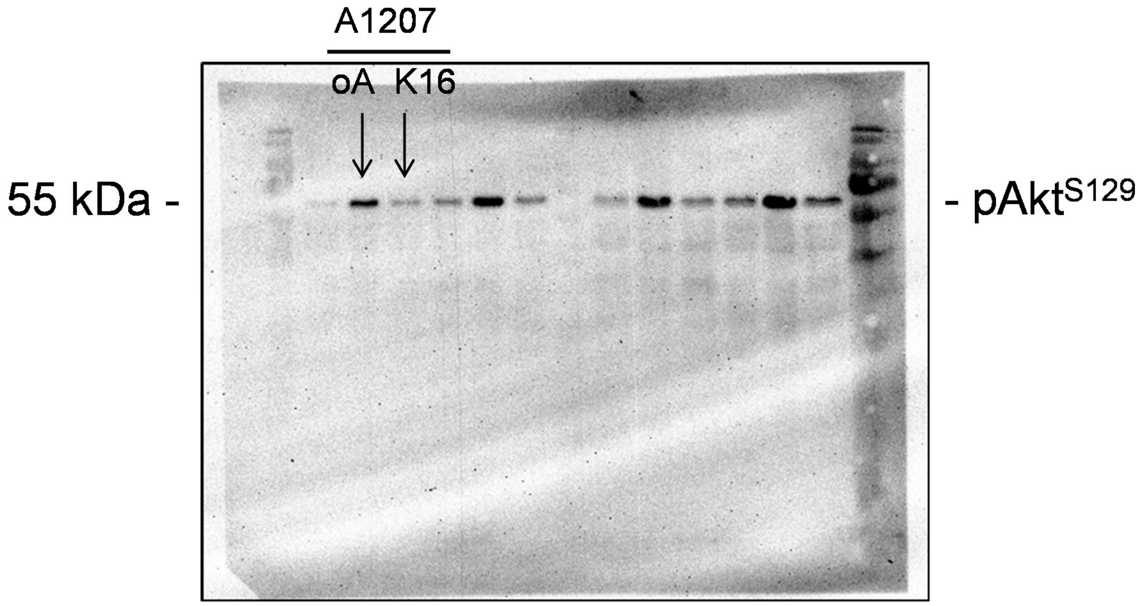

Figure S13. CK2 inhibition reduces NG2 expression in human GBM cell lines. A1207 wild type and CK2α KO cells were lysed and the expression of pAkt^{S129} was analyzed by western blot.

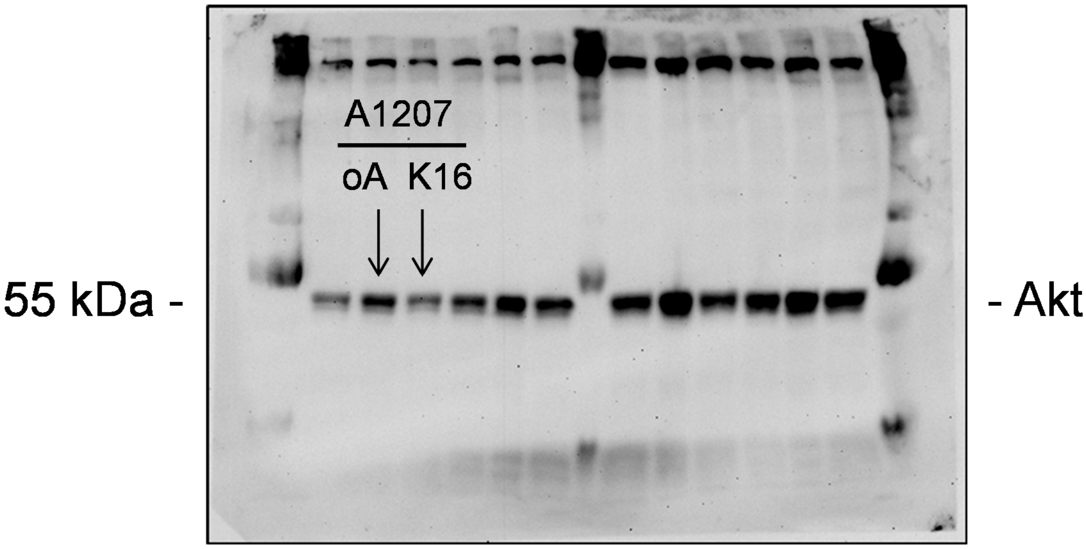

Figure S14. CK2 inhibition reduces NG2 expression in human GBM cell lines. A1207 wild type and CK2α KO cells were lysed and the expression of Akt was analyzed by western blot.

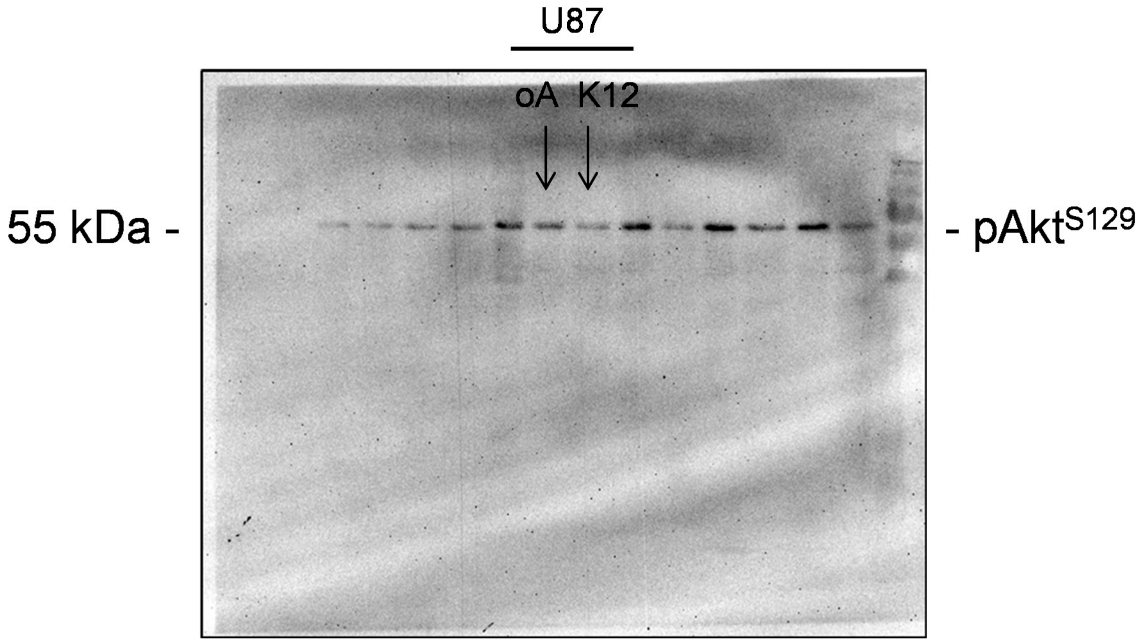

Figure S15. CK2 inhibition reduces NG2 expression in human GBM cell lines. U87 wild type and CK2α KO cells were lysed and the expression of pAkt^{S129} was analyzed by western blot.

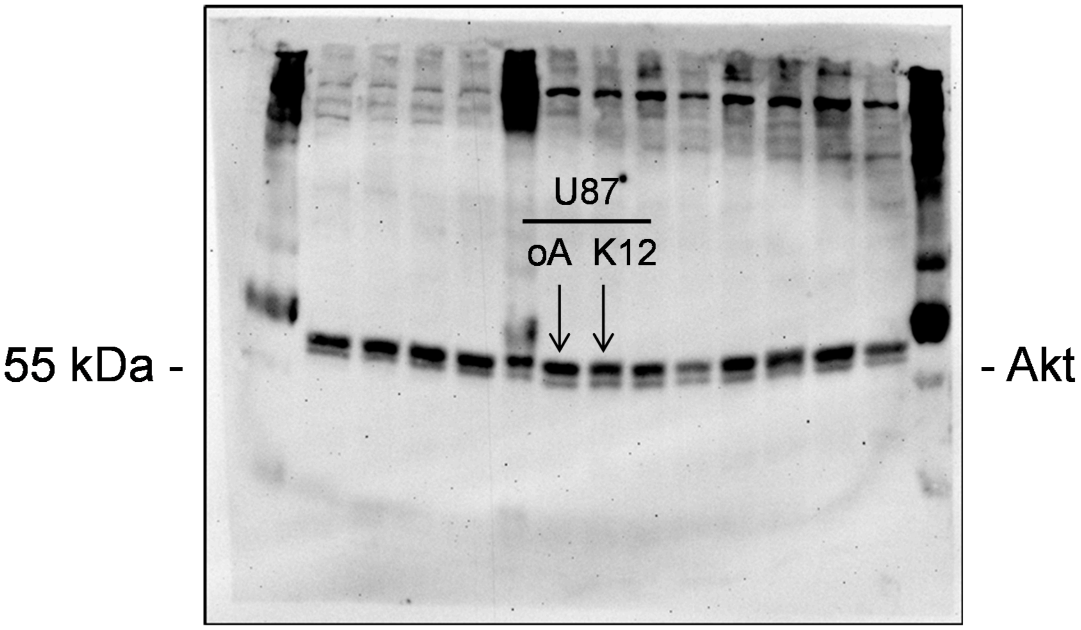

Figure S16. CK2 inhibition reduces NG2 expression in human GBM cell lines. U87 wild type and CK2α KO cells were lysed and the expression of Akt was analyzed by western blot.

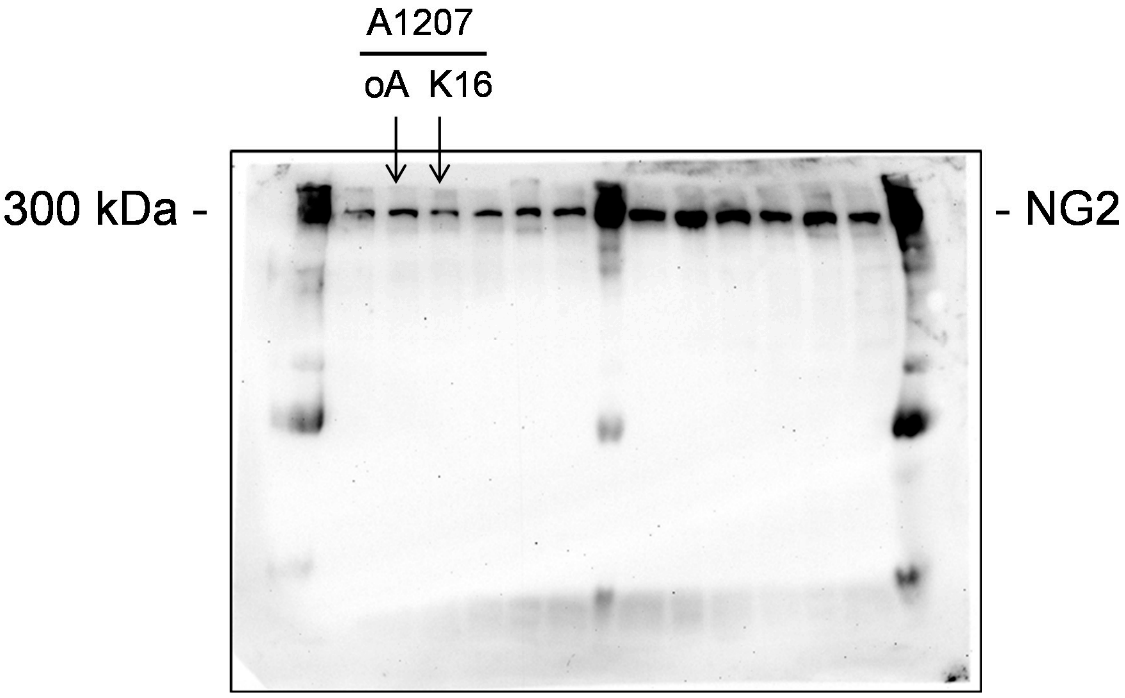

Figure S17. CK2 inhibition reduces NG2 expression in human GBM cell lines. A1207 wild type and CK2α KO cells were lysed and the expression of NG2 was analyzed by western blot.

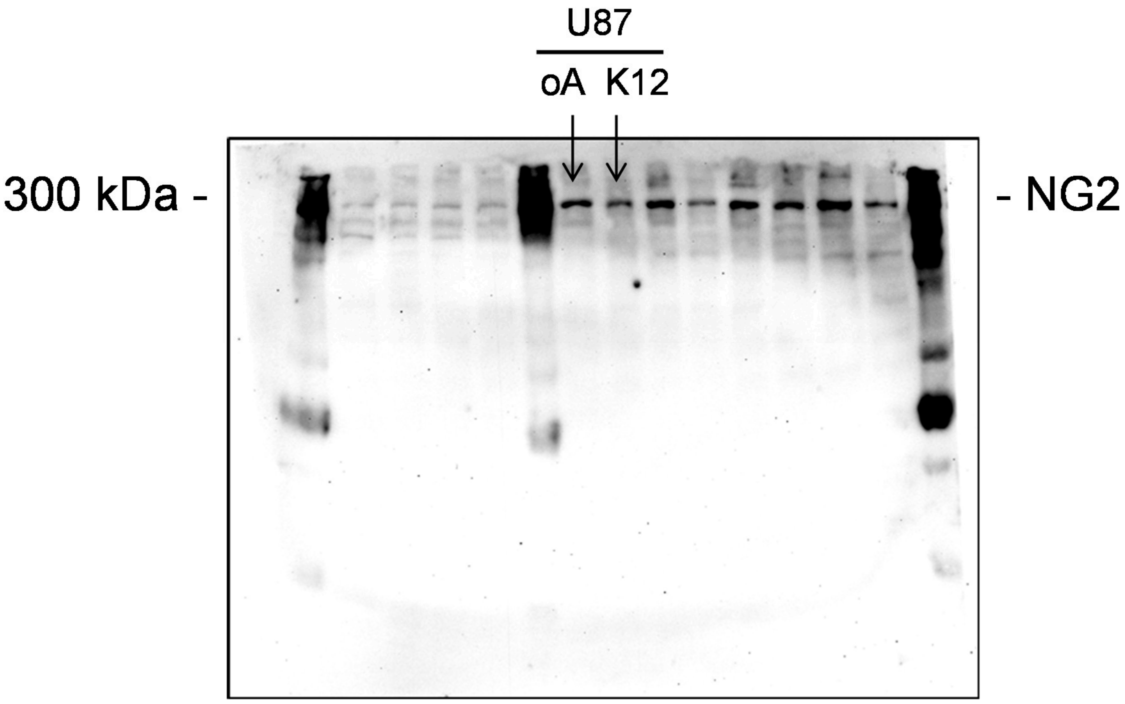

Figure S18. CK2 inhibition reduces NG2 expression in human GBM cell lines. U87 wild type and CK2α KO cells were lysed and the expression of NG2 was analyzed by western blot.

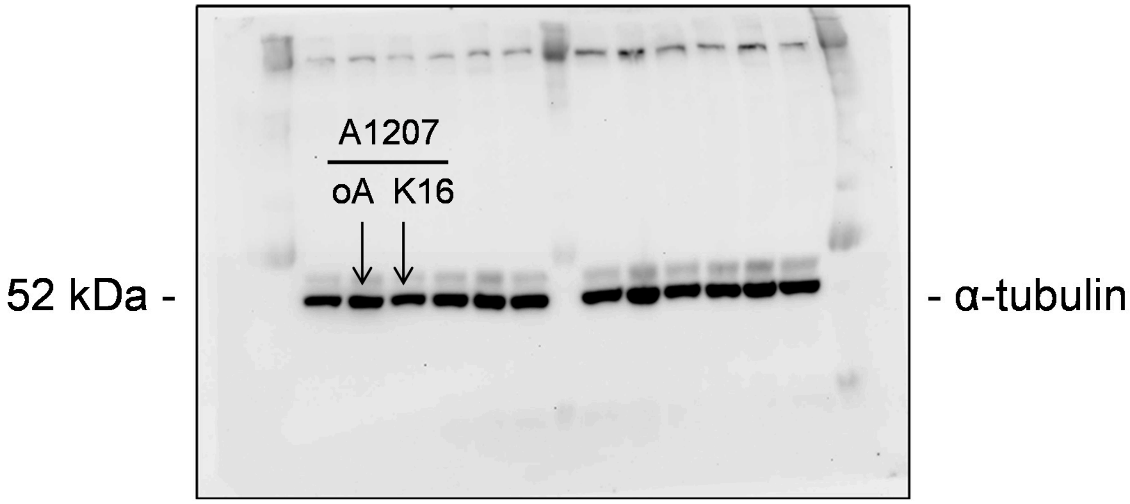

Figure S19. CK2 inhibition reduces NG2 expression in human GBM cell lines. A1207 wild type and CK2α KO cells were lysed and the expression of α-tubulin (as loading control) was analyzed by western blot.

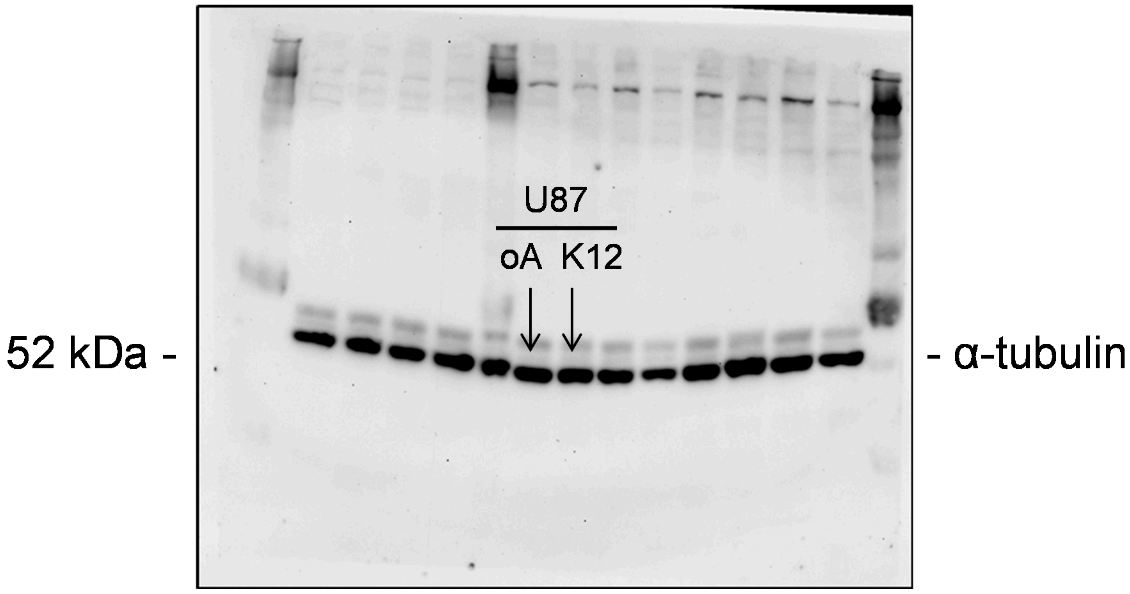

Figure S20. CK2 inhibition reduces NG2 expression in human GBM cell lines. U87 wild type and CK2α KO cells were lysed and the expression of α-tubulin (as loading control) was analyzed by western blot.

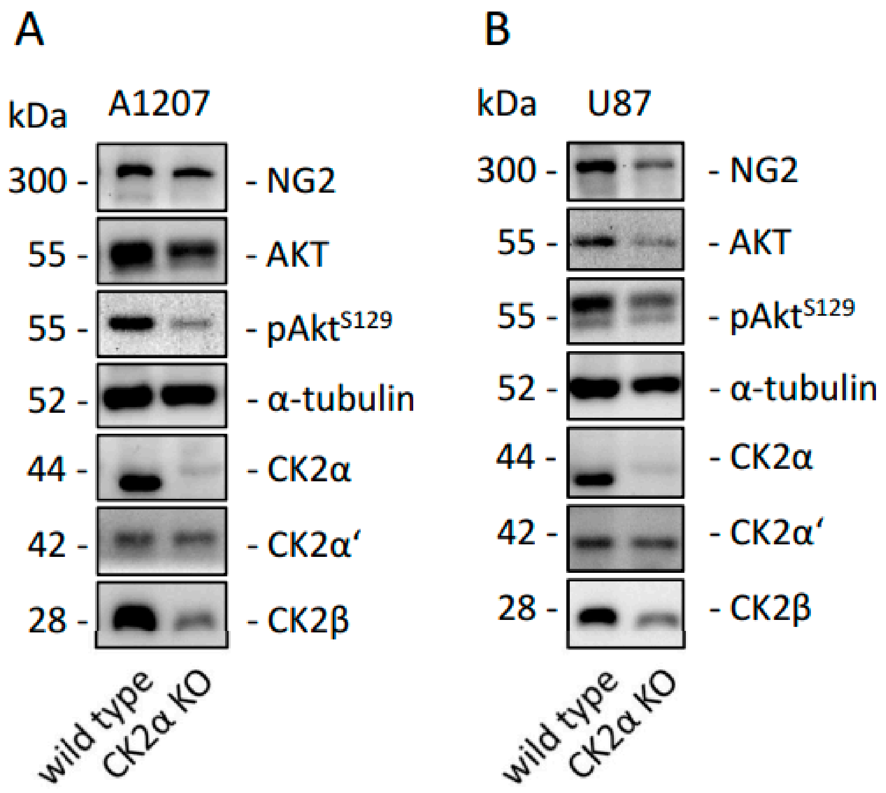

Figure S21. (**A** and **B**) A1207 (A) and U87 (B) wild type and CK2*α* KO cells were lysed and the expression of NG2, Akt, pAkt^{S129}, CK2*α*, CK2*α*′, CK2β and *α*-tubulin (as loading control) was analyzed by Western blot.

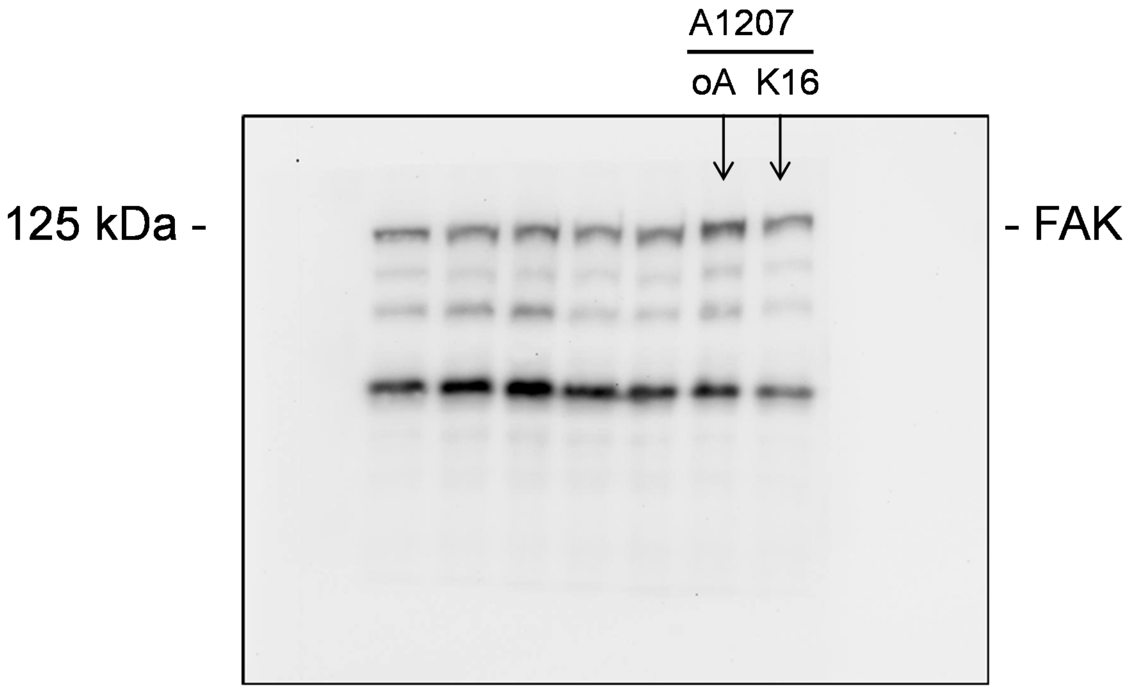

Figure S22. CK2 inhibition reduces NG2 expression in human GBM cell lines. A1207 wild type and CK2*α* KO cells were lysed and the expression of FAK was analyzed by western blot.

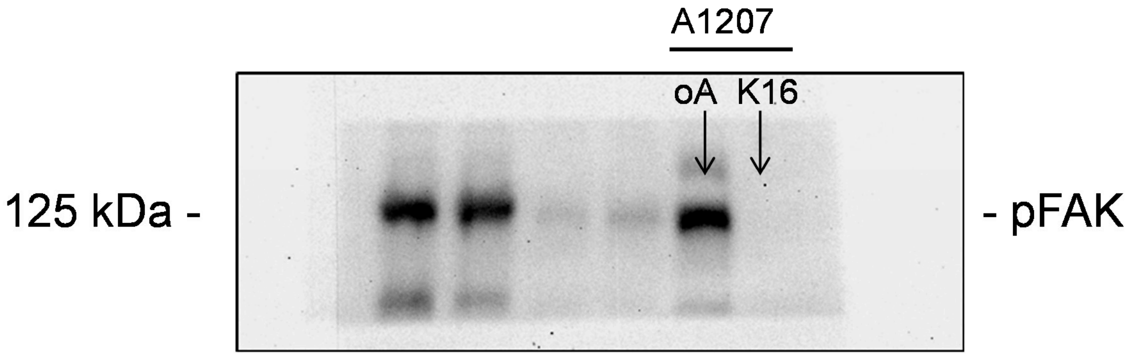

Figure S23. CK2 inhibition reduces NG2 expression in human GBM cell lines. A1207 wild type and CK2α KO cells were lysed and the expression of pFAK was analyzed by western blot.

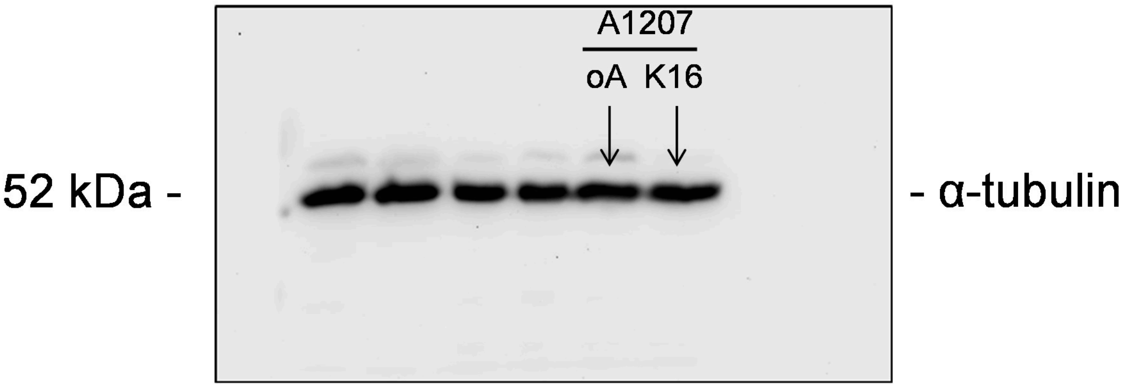

Figure S24. CK2 inhibition reduces NG2 expression in human GBM cell lines. A1207 wild type and CK2α KO cells were lysed and the expression of α-tubulin (as loading control) was analyzed by western blot.

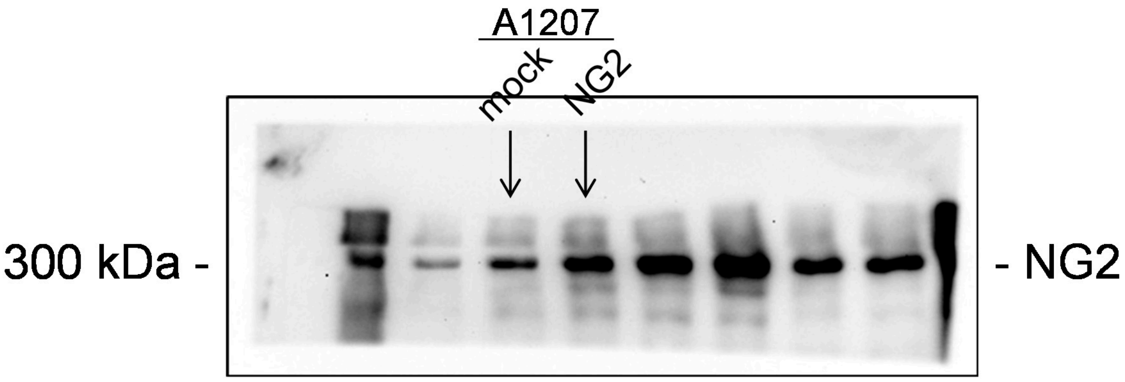

Figure S25. CK2 inhibition reduces the migratory capacity of NG2-positive GBM cell lines. A1207 were transfected with mock (pEF6 vector) or NG2 plasmid and incubated for 48 h. Expression of NG2 was analyzed by western blot.

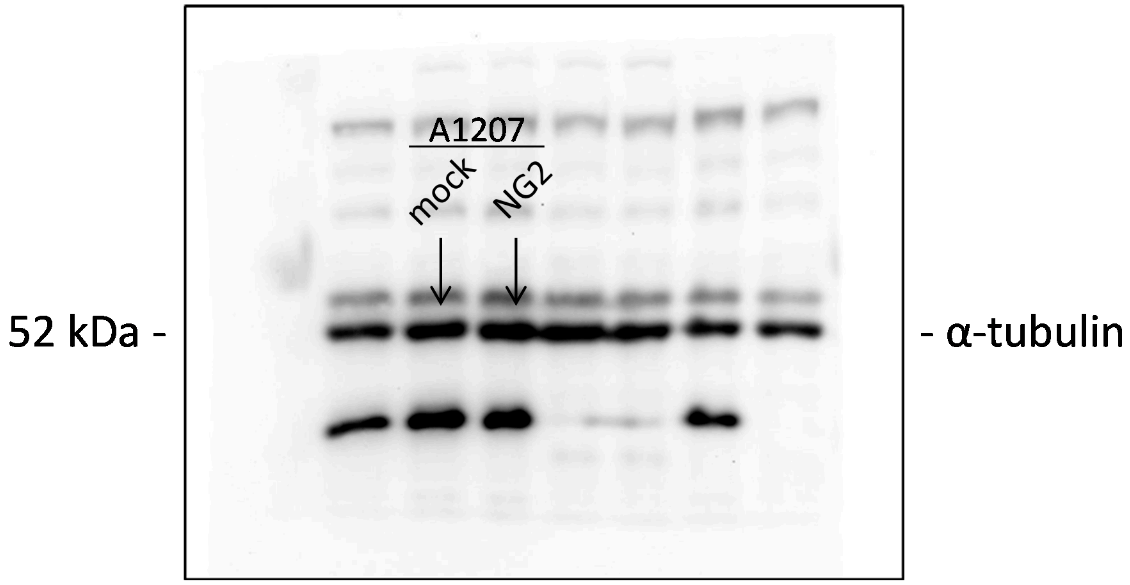

Figure S26. CK2 inhibition reduces the migratory capacity of NG2-positive GBM cell lines. A1207 were transfected with mock (pEF6 vector) or NG2 plasmid and incubated for 48 h. Expression of *α*-tubulin (as loading control) was analyzed by western blot.

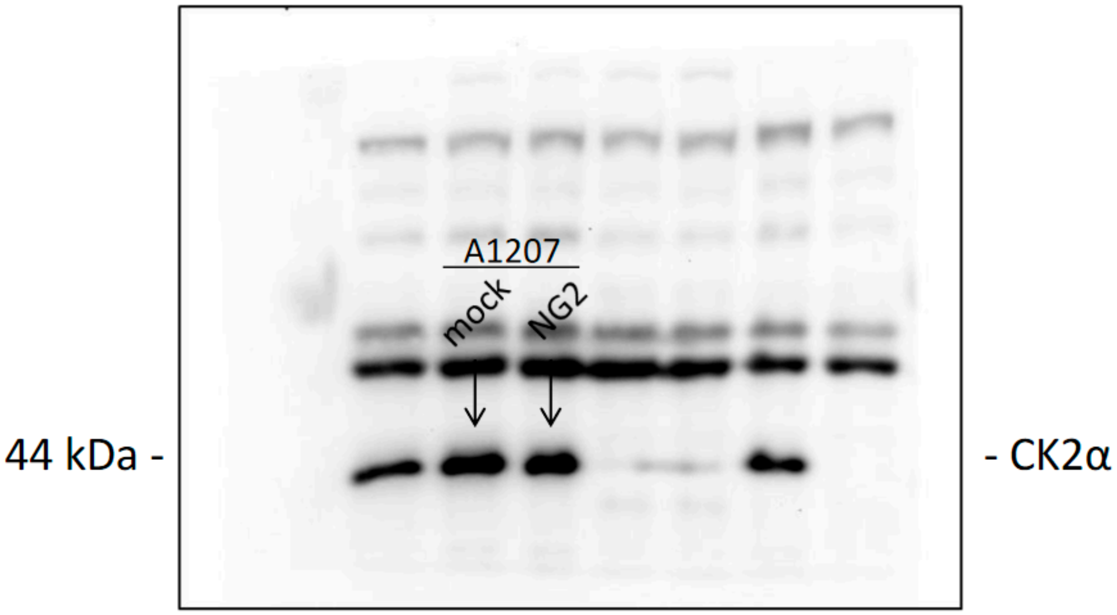

Figure S27. CK2 inhibition reduces the migratory capacity of NG2-positive GBM cell lines. A1207 were transfected with mock (pEF6 vector) or NG2 plasmid and incubated for 48 h. Expression of CK2*α* was analyzed by western blot.

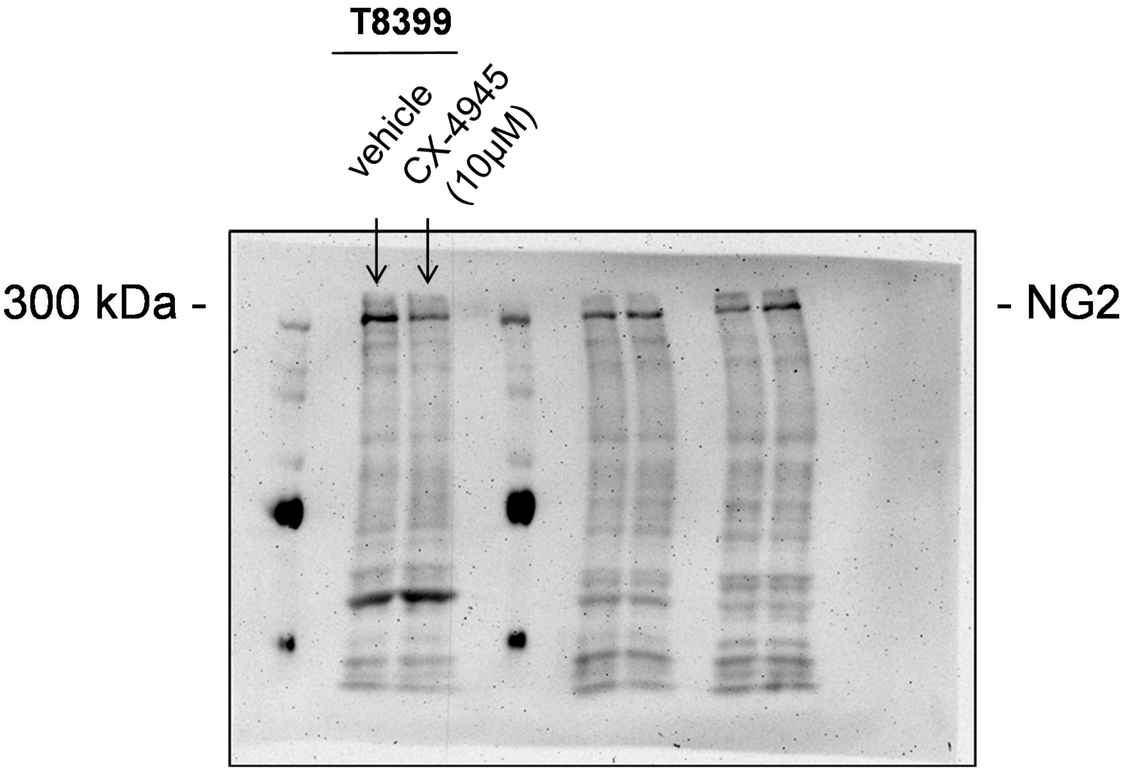

Figure S28. CK2 inhibition reduces NG2 expression in patient-derived GBM cells. Patient-derived GBM cells (T8399) were treated with vehicle (DMSO) or CX-4945 (10 µM) for 72 h. Subsequently, the cells were lysed and the expression of NG2 was analyzed by western blot.

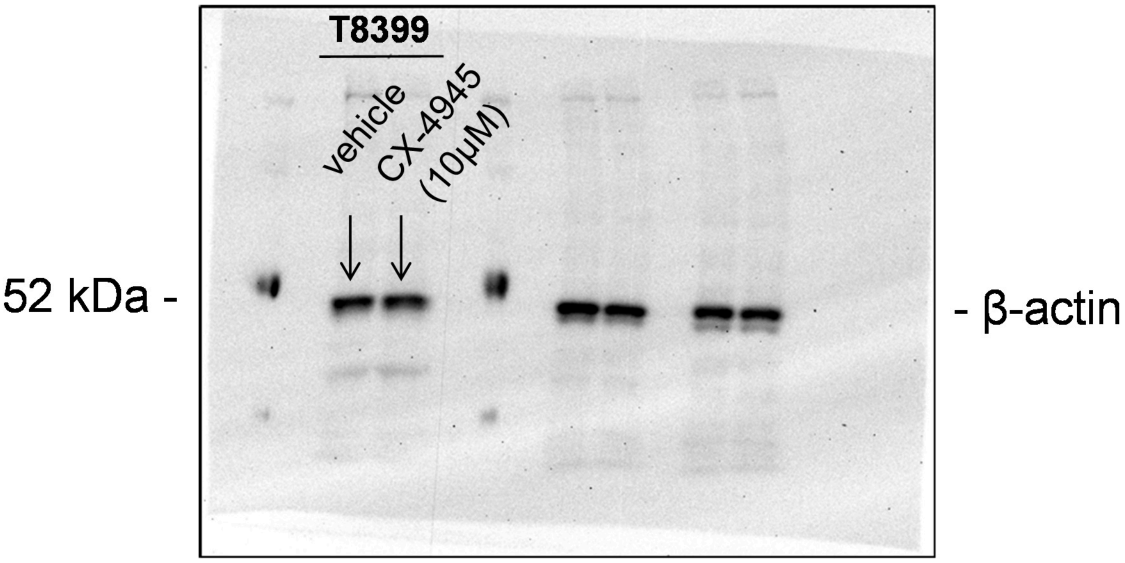

Figure S29. CK2 inhibition reduces NG2 expression in patient-derived GBM cells. Patient-derived GBM cells (T8399) were treated with vehicle (DMSO) or CX-4945 (10 µM) for 72 h. Subsequently, the cells were lysed and the expression of β-actin (as loading control) was analyzed by western blot.

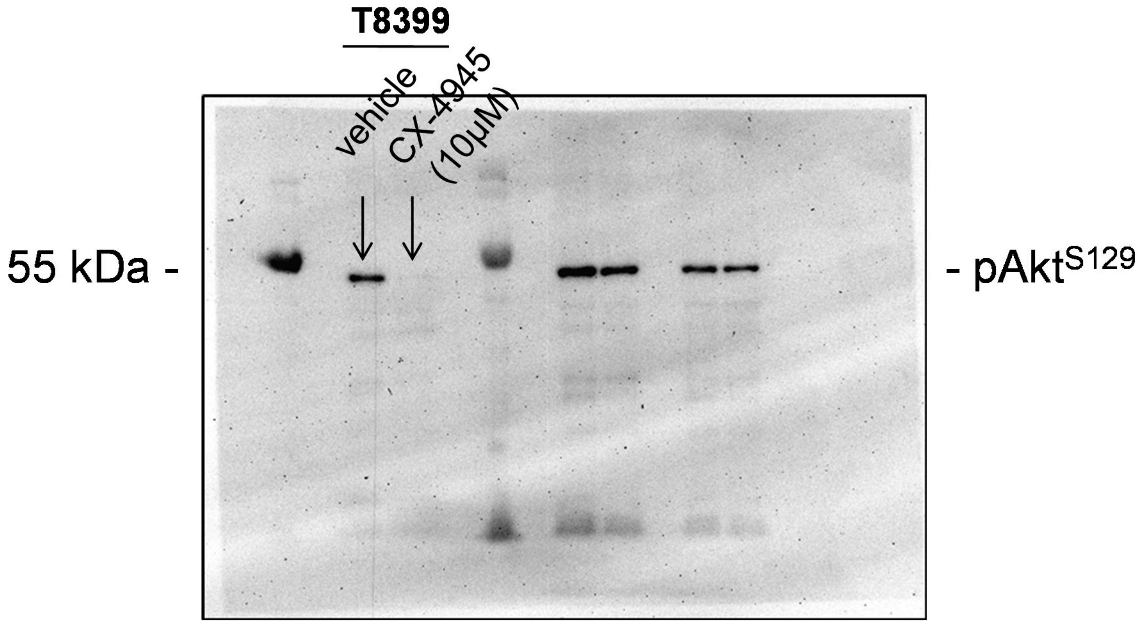

Figure S30. CK2 inhibition reduces NG2 expression in patient-derived GBM cells. Patient-derived GBM cells (T8399) were treated with vehicle (DMSO) or CX-4945 (10 µM) for 72 h. Subsequently, the cells were lysed and the expression of $pAkt^{S129}$ was analyzed by western blot.

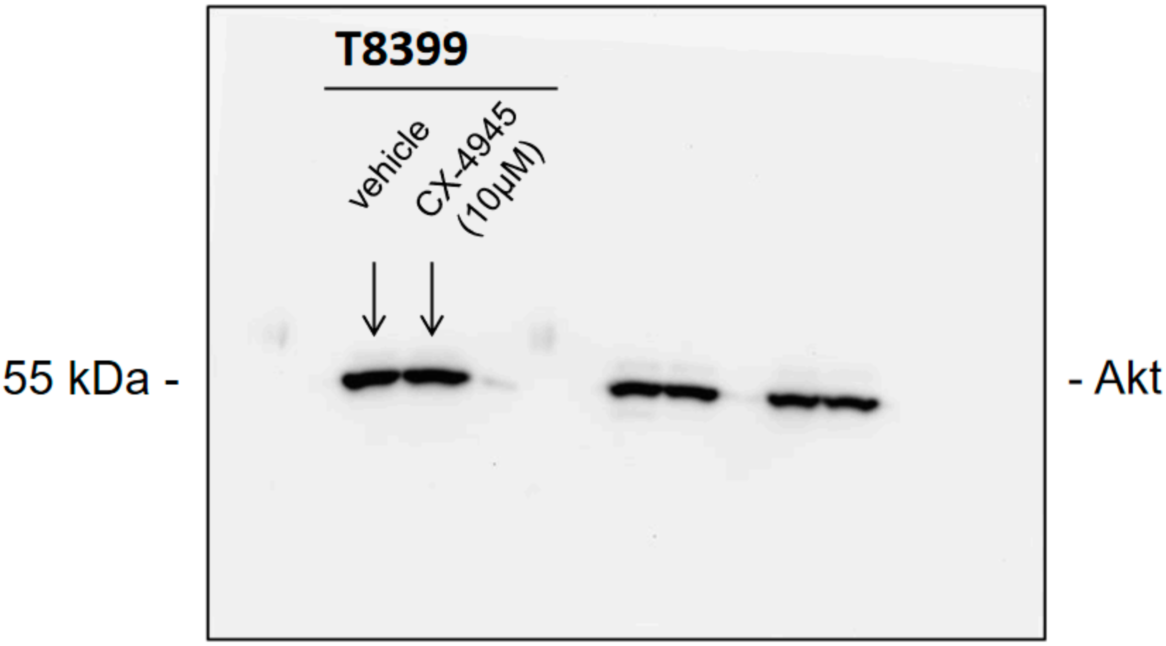

Figure S31. CK2 inhibition reduces NG2 expression in patient-derived GBM cells. Patient-derived GBM cells (T8399) were treated with vehicle (DMSO) or CX-4945 (10 µM) for 72 h. Subsequently, the cells were lysed and the expression of Akt was analyzed by western blot.

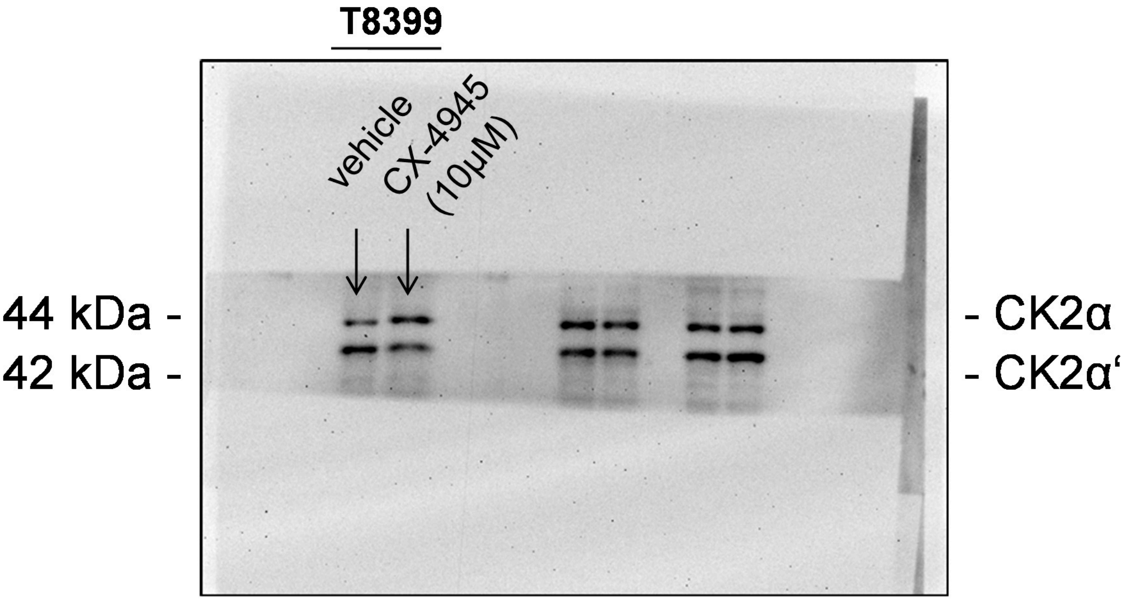

Figure S32. CK2 inhibition reduces NG2 expression in patient-derived GBM cells. Patient-derived GBM cells (T8399) were treated with vehicle (DMSO) or CX-4945 (10 µM) for 72 h. Subsequently, the cells were lysed and the expression of CK2α, CK2α′ was analyzed by western blot.

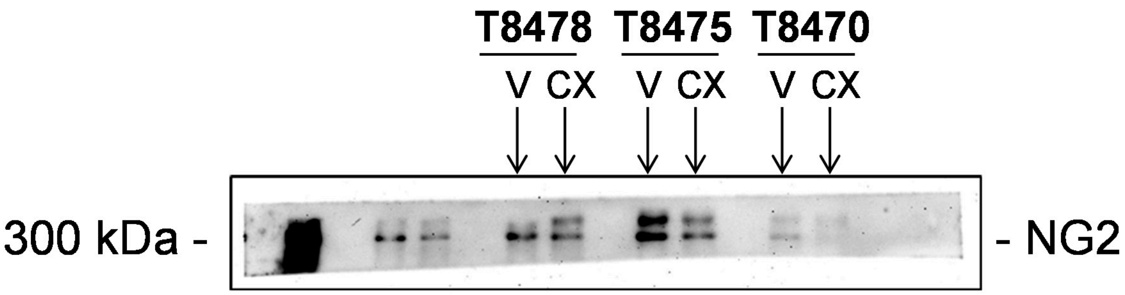

Figure S33. CK2 inhibition reduces NG2 expression in patient-derived GBM cells. Patient-derived GBM cells (T8478, T8475 and T8470) were treated with vehicle (DMSO) or CX-4945 (10 µM) for 72 h. Subsequently, the cells were lysed and the expression of NG2 was analyzed by western blot.

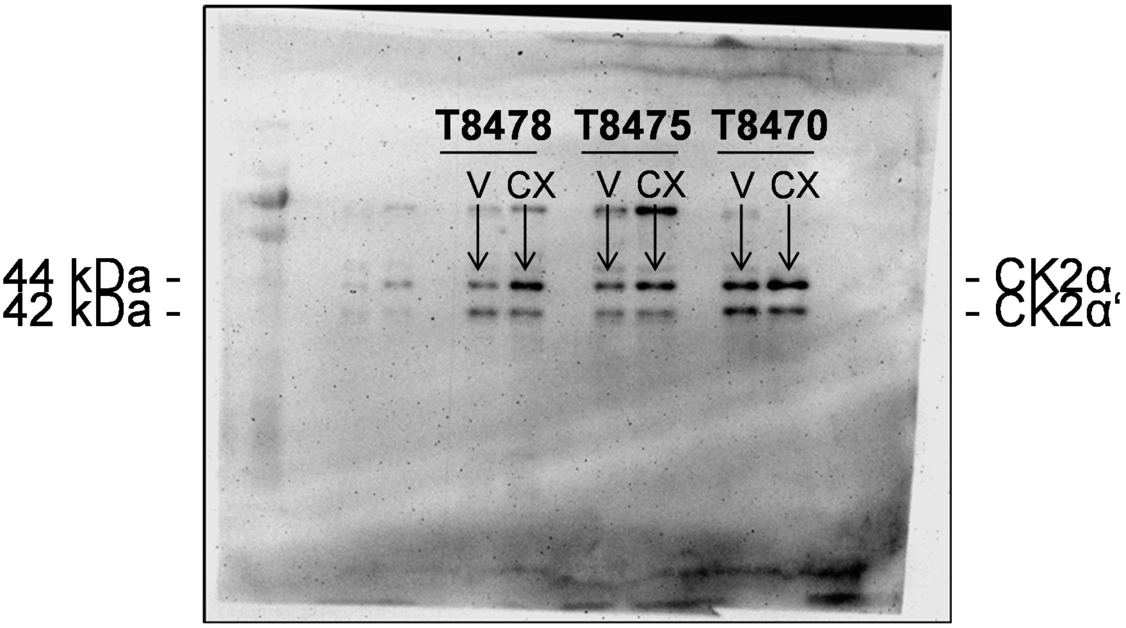

Figure S34. CK2 inhibition reduces NG2 expression in patient-derived GBM cells. Patient-derived GBM cells (T8478, T8475 and T8470) were treated with vehicle (DMSO) or CX-4945 (10 μM) for 72 h. Subsequently, the cells were lysed and the expression of CK2α and CK2α' was analyzed by western blot.

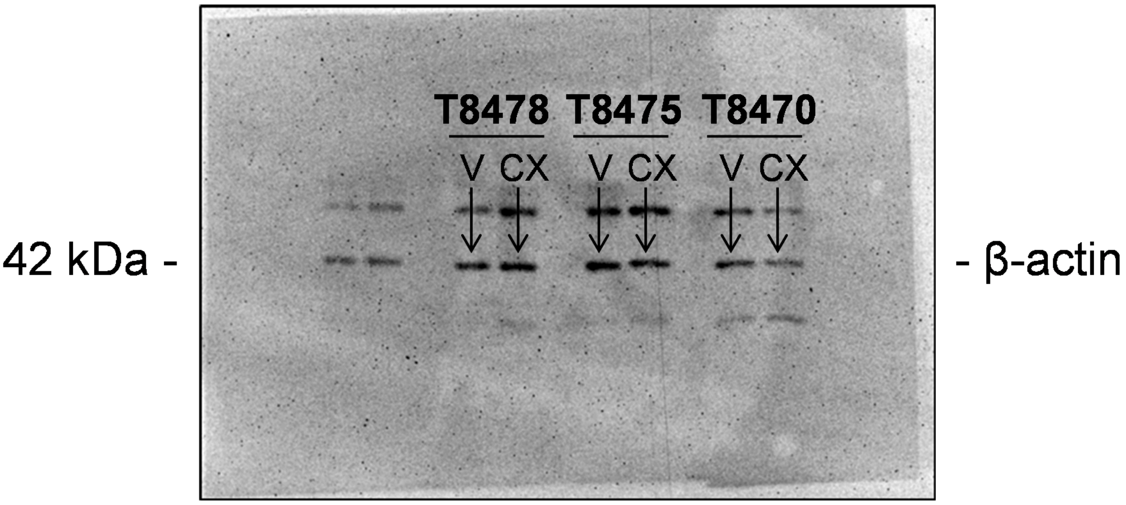

Figure S35. CK2 inhibition reduces NG2 expression in patient-derived GBM cells. Patient-derived GBM cells (T8478, T8475 and T8470) were treated with vehicle (DMSO) or CX-4945 (10 μM) for 72 h. Subsequently, the cells were lysed and the expression of β-actin (as loading control) was analyzed by western blot.

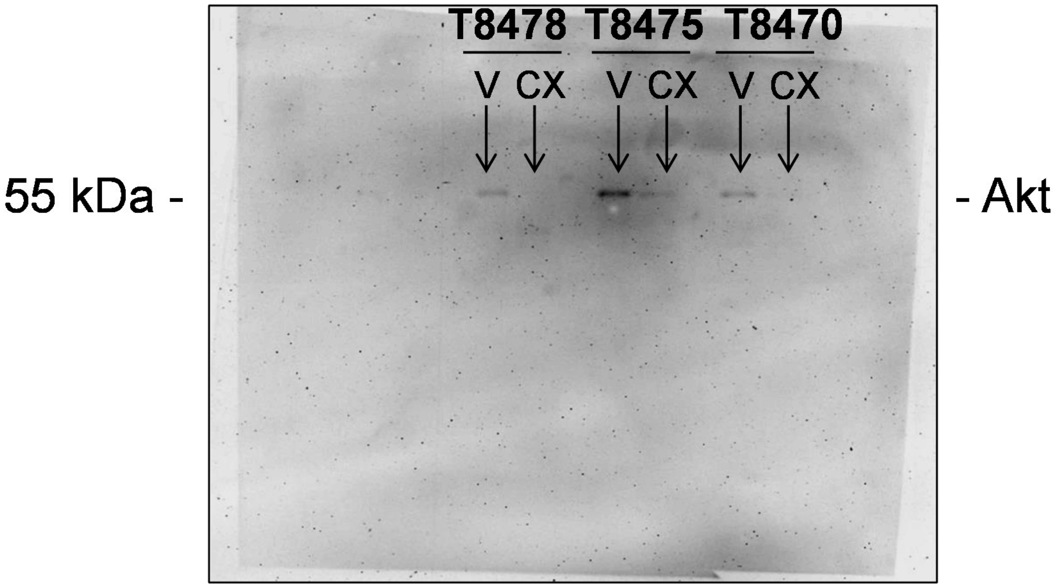

Figure S36. CK2 inhibition reduces NG2 expression in patient-derived GBM cells. Patient-derived GBM cells (T8478, T8475 and T8470) were treated with vehicle (DMSO) or CX-4945 (10 μM) for 72 h. Subsequently, the cells were lysed and the expression of Akt was analyzed by western blot.

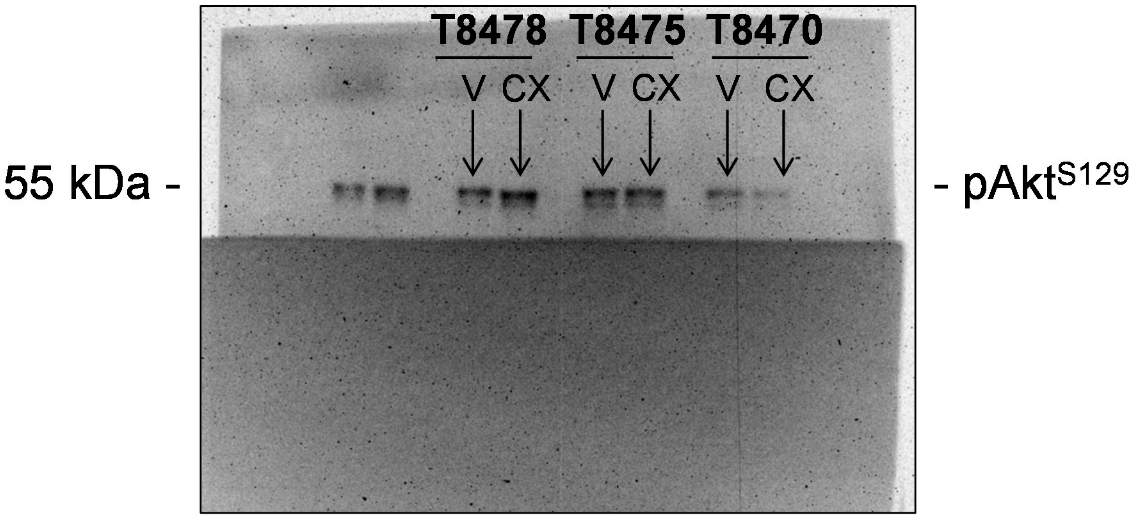

Figure S37. CK2 inhibition reduces NG2 expression in patient-derived GBM cells. Patient-derived GBM cells (T8478, T8475 and T8470) were treated with vehicle (DMSO) or CX-4945 (10 μM) for 72 h. Subsequently, the cells were lysed and the expression of $pAkt^{S129}$ was analyzed by western blot.

© 2021 by the authors. Licensee MDPI, Basel, Switzerland. This article is an open access article distributed under the terms and conditions of the Creative Commons Attribution (CC BY) license (http://creativecommons.org/licenses/by/4.0/).
